# Development of a movement-based *in vitro* screening assay for the identification of new anti-cestodal compounds

**DOI:** 10.1371/journal.pntd.0005618

**Published:** 2017-05-17

**Authors:** Dominic Ritler, Reto Rufener, Heinz Sager, Jacques Bouvier, Andrew Hemphill, Britta Lundström-Stadelmann

**Affiliations:** 1 Institute of Parasitology, Vetsuisse Faculty, University of Berne, Berne, Switzerland; 2 Elanco Animal Health, Basel, Switzerland; University of Würzburg, GERMANY

## Abstract

Intestinal cestodes are infecting millions of people and livestock worldwide, but treatment is mainly based on one drug: praziquantel. The identification of new anti-cestodal compounds is hampered by the lack of suitable screening assays. It is difficult, or even impossible, to evaluate drugs against adult cestodes *in vitro* due to the fact that these parasites cannot be cultured in microwell plates, and adult and larval stages in most cases represent different organisms in terms of size, morphology, and metabolic requirements. We here present an *in vitro*-drug screening assay based on *Echinococcus multilocularis* protoscoleces, which represent precursors of the scolex (hence the anterior part) of the adult tapeworm. This movement-based assay can serve as a model for an adult cestode screen. Protoscoleces are produced in large numbers in Mongolian gerbils and mice, their movement is measured and quantified by image analysis, and active compounds are directly assessed in terms of morphological effects. The use of the 384-well format minimizes the amount of parasites and compounds needed and allows rapid screening of a large number of chemicals. Standard drugs showed the expected dose-dependent effect on movement and morphology of the protoscoleces. Interestingly, praziquantel inhibited movement only partially within 12 h of treatment (at concentrations as high as 100 ppm) and did thus not act parasiticidal, which was also confirmed by trypan blue staining. Enantiomers of praziquantel showed a clear difference in their minimal inhibitory concentration in the motility assay and (R)-(-)-praziquantel was 185 times more active than (S)-(-)-praziquantel. One compound named MMV665807, which was obtained from the open access MMV (Medicines for Malaria Venture) Malaria box, strongly impaired motility and viability of protoscoleces. Corresponding morphological alterations were visualized by scanning electron microscopy, and demonstrated that this compound exhibits a mode of action clearly distinct from praziquantel. Thus, MMV665807 represents an interesting lead for further evaluation.

## Author summary

Tapeworms (cestodes) are a medically important group of helminths that infect humans and animals all around the globe. The clinical signs caused by intestinal infection with adult cestodes are mostly mild, in contrast to the more severe disease symptoms inflicted by infection with the tissue-dwelling larval stages of the same species. Praziquantel is the main drug in use against intestinal cestode infections. Development of resistance and treatment failures have been reported in trematodes, and are expected to become a problem in the future also in the case of cestode infections. Therefore, new treatment options against intestinal helminths are needed. To date, there is no *in vitro*-based whole-organism screening assay available that allows screening of candidate drugs with potential activity against adult cestodes. We established and characterized of a screening assay in 384-well format, which serves as a model for adult stage parasites by using *Echinococcus multilocularis* protoscoleces and their loss of motility as a read-out. This novel assay showed that drugs with known activity against adult cestodes inhibited motility of protoscoleces. The movement-based assay identified MMV665807 as a novel compound with profound activity against protoscoleces, and potentially also adult cestodes. Light- and electron microscopical assessments of protoscoleces treated with praziquantel and MMV665807 point towards different modes of action of the two drugs.

## Introduction

Helminths are separated into the two major phyla of nematodes (roundworms) and platyhelminths (flatworms), including trematodes and cestodes, and they are important causes of disease in humans as well as animals. An estimated one billion people are infected with at least one helminth in developing countries of Africa, Asia and America [1]. Infection of livestock by helminths, small ruminants in particular, has an enormous economic impact on productivity in farming [2]. Despite the large number of infected individuals and enormous economic losses due to helminth infections in animals, there are still not many drugs registered for their treatment [1]. Present efforts ongoing to discover new anthelminthic drugs are focused on gastrointestinal nematodes, schistosomes and filariae [3], as they comprise the highest prevalences. However, most of the adult stages of trematodes, and all cestodes, are not being considered in the current drug screening efforts. Intestinal cestodes might be considered as parasites of lower relevance as they usually cause few clinical signs, but they are of high relevance as source of infection of diseases caused by the larval stages of these parasites [4,5].

For the treatment of nematodes, a variety of drugs are in use, and new ones have been introduced to the market recently. However, spread of resistance is a major problem in the veterinary sector [6]. For treatment of cestode and trematode infections, praziquantel (PZQ) is the drug of choice against most species [7]. For cestode-treatment, the alternatives available are epsiprantel that is exclusively applied in animals, and niclosamide, whose marketing status is currently discontinued [4,8]. PZQ is generally very well tolerated, even though it tastes bitter. It induces only mild adverse reactions, but rare events of allergy and hypersensitivity reaction have been described [7]. However, there is increasing evidence on resistance of schistosomes against PZQ [9] and treatment failures of PZQ against *Taenia saginata* are described as well [10]. A major problem is that PZQ is the only drug in use against many platyhelminths and mass drug administrations all over the globe might select for resistant platyhelminth strains in the future. In helminths, as compared to for example bacteria, resistance development takes more time as their generation time is much longer. Nevertheless, drug resistance is already a major problem for many diseases caused by nematodes and trematodes, and it will be only a question of time until resistance to PZQ spreads also to cestodes [11,12]. Therefore, it is of crucial importance to search for new anthelminthic drugs, including compounds against platyhelminths.

Whole-organism screening is still the most widely accepted method for anti-parasitic drug discovery, despite the fact that target-based screening approaches have been widely introduced [3]. However, this approach is challenging as it relies on a profound knowledge of parasite life-stages and their biology, the availability of *in vitro* culture techniques, and reliable methods for compound efficacy assessment [3]. *Caenorhabditis elegans* is a frequently used model worm for whole-organism *in vitro* screenings, but over the last decades also other models have been successfully established. Classically, the viability and morphology of whole parasites upon treatment is assessed by light microscopy, which harbors drawbacks such as time-consuming and subjective evaluation procedures, and automatization is not possible. However, no specialized equipment is required [13]. Other assays include dyes that indicate viability or loss of viability such as trypan blue and eosin or even fluorescent stains, but nevertheless, these are low-throughput methods and they represent only indirect indicators of viability [14,15]. For objective larger-scale screenings, new methods were implemented over the last decades, such as MTT (3-(4,5-Dimethylthiazol-2-yl)-2,5-diphenyltetrazolium bromide) assay, alamar Blue assay or fluorescent labeling that allow an automated readout [3,14].

The observation that anthelminthics reduce larval motility in nematodes led to the development of a motility-based assay for assessing the effects of certain compounds [16]. Non-image-based methods to measure nematode motility include measurements of the fluctuations in electrical currents by xCELLigence [17] or isothermal microcalorimetry [18]. In addition, image-based methods have been introduced, such as the Parallel Worm Tracker [19], the WormAssay [20], the WormScan [21], the Worminator [22] or the whole-organism screening by Preston et al [23].Adult cestodes have so far been excluded from *in vitro* screening, since for most of these parasites *in vitro* culture methods have not been established, or they are too large to be cultured in tissue culture devices suitable for screening assays.

Adult *E*. *multilocularis* tapeworms live in the small intestine of final hosts, such as foxes and dogs, and they release infectious eggs in the faeces of these hosts. Upon ingestion of eggs, intermediate hosts, such as rodents and small mammals, get infected with the parasite and a multivesicular metacestode will develop mainly in their livers. After 2–4 months, brood capsules with protoscoleces will form within these metacestodes. Once a final host feeds upon an intermediate hosts, protoscoleces get ingested as well, and they will develop into adult tapeworms in the intestine of these hosts [24]. The fox tapeworm *Echinococcus multilocularis* has become an important model for the study of cestodes, since the genome has been sequenced and corresponding data is publically available, including also the close relative *E*. *granulosus* [25,26], advanced molecular tools have been developed for the study of the disease-causing metacestode stage [27,28], and metacestode *in vitro* drug screening assays have been implemented [29–31]. However, it is known that drugs with activity against larval stages are not necessarily active against adult stages and vice versa [3,14]. Albendazole is the drug of choice against alveolar echinococcosis caused by *E*. *multilocularis* metacestodes, but the drug is ineffective against adults. On the other hand, PZQ is the most widely used drug against adult cestodes, but it is not active against metacestodes. Adult *E*. *multilocularis* worms have not been studied extensively, due to high risk of infection for the experimenter and lack of suitable laboratory models. There are a number of studies on protoscolicidal substances for the treatment of *E*. *granulosus* infections. However, none of these have considered protoscoleces as a potential model for adult tapeworms. Protoscoleces are easily generated and purified in large numbers, they are relatively small (150–350 μM in length), move actively and they represent precursors of adult tapeworms. Thus, we describe here an *in vitro* drug screening assay that is easy to perform, inexpensive, and which is based on the semi-automated, quantitative assessment of *E*. *multilocularis* protoscolex motility. In addition, we also compare the method of motility assessment to the classical trypan blue staining method.

## Materials and methods

If not stated otherwise, all chemicals were purchased from Sigma (St. Louis, MO, USA). Dulbecco’s modified Eagle medium (DMEM) and fetal calf serum (FCS) were from Biochrom (Berlin, Germany). Plastic ware was from Sarstedt (Nümbrecht, Germany). All drugs tested in the present study were obtained from Elanco (St. Aubin, Switzerland) and they were all delivered as stocks of 10 g/L in DMSO. The drugs applied for experiments of Supplementary S3 Fig were purchased from Sigma, and prepared as stocks of 10 g/L as well.

### Parasite material

*E*. *multilocularis* protoscoleces were extracted under sterile conditions from *E*. *multilocularis* metacestodes kindly provided from strain maintenance surplus by Dr. R. Fiechter (permission number ZH139/2015, Institute of Parasitology, Zürich, Switzerland). The parasite originated from naturally infected monkeys from the German primate center, and they had been under *in vivo* passage in Mongolian gerbils for up to five years. The protocol described by Brehm et al was applied to purify protoscoleces from metacestodes with minor changes [32]. In short, the metacestode material was pressed through a tea sieve, washed out with PBS and further broken mechanically by vigorous shaking in a QIAGEN tissue lyzer for 10 minutes (8 shakes per second). The resulting suspension was passed through a sieve with 250 μM mesh size. The flow through was then passed through a second sieve with 50 μM mesh size and thoroughly washed with PBS. The protoscoleces that were collected in the 50 μM sieve were transferred into a 50 mL tube and washed repeatedly with PBS until all remaining vesicle tissue was removed and the protoscoleces sedimented fast. For further purification, the protoscoleces were given into a petri dish (14 cm diameter) containing 70 mL DMEM with 10% FCS. The dish was moved in circles so the protoscoleces could be collected in the centre of the plate and they were again washed in PBS before being immediately subjected to activation (see below).

### Protoscolex activation

The standard procedures for evagination of protoscoleces are based on incubation in pepsin (0.5 mg/mL), pH 2.0 for 3 hours at 37°C [33–35], or pepsin (0.05%), pH 2.0 for 30 minutes and subsequent incubation in 0.2% Na-taurocholate for 3 hours at 37°C [36,37]. In order to simplify this method for a subsequent motility-based screening, we incubated freshly extracted protoscoleces also for 3 hours at various concentrations of DMSO (0, 1, 2.5, 5, 10, and 20%) in DMEM, including 10% FCS. All incubations were done in triplicates. Subsequently, the protoscoleces were washed in PBS and were allowed to recover overnight in DMEM with 10% FCS at 37°C and 5% CO_2_. Thereafter, the numbers of invaginated and evaginated protoscoleces were counted manually under the light microscope within one field of vision (40 x magnification, > 170 protoscoleces per view) and the percentage of evaginated protoscoleces was determined. Averages and standard deviations were calculated in Microsoft Office Excel 2010. The experiment was repeated three times.

Based on the results obtained above, a standardized activation protocol was established: a maximum of 25’000 protoscoleces per well were seeded in a 6-well pate in DMEM containing 10% FCS and 10% DMSO, at 37°C, and 5% CO_2_ for 3 hours. Thereafter, the protoscoleces were washed in PBS twice at room temperature and incubated in DMEM with 10% FCS and antibiotics (100 U/mL penicillin, 100 μg/mL streptomycin) at 37°C, and 5% CO_2_, overnight. For all subsequent manipulations, pipettes and tubes were pre-rinsed with FCS in order to avoid sticking of protoscoleces to the tubing walls.

### Image-based motility assessment

Photomicrographs of protoscoleces were taken in a live cell imaging system (Nikon TE2000E microscope connected to a Hamatsu ORCA ER camera) with the software NIS Elements Version 4.40 and the additional module JOBS (JOBS program given in S1 File). For motility measurement, two separate images were taken of each well at a 10 seconds interval and at 40 times magnification. Motility was assessed at various time points (1, 6, 12, 18, and 24 h) after addition of compounds. The motility index for each well, resulting from differences in pixels within the 10 seconds interval, was calculated with a pixel grey value threshold of 230 in ImageJ version 1.49 (the respective Macro including details of calculation is given in S2 File). Averages of movement indices (excluding the minimal and maximal values of each of the six replicas) and respective standard deviations were calculated in Microsoft Office Excel 2010.

### Number to motility ratio experiment

In order to check the correlation of number and movement of protoscoleces in the 384-well format, various numbers of protoscoleces (1 to 58) were tested. Protoscoleces were seeded in a total of 20 μL of DMEM without phenol red, including 10% FCS and 1% DMSO, in a 384-well plate (order nb. 788095–128, Greiner bio-one, via Huberlab, Aesch, Switzerland) and sealed by a pressure sensitive seal (order nb. 7676–070, Greiner bio-one, via Huberlab). The total motility corresponding to each number of protoscoleces was assessed as described above, but without normalization and the correlation was determined in R (version 3.3.0).

### Assessment of optimal temperature for motility assay

To determine the optimal temperature for the motility assay, the same general protocol was used as in the number to motility ratio experiment (see above): 20–30 protoscoleces per well were seeded in a total of 20 μL of DMEM without phenol red, including 10% FCS and 1% DMSO. Automated seeding was performed by application of a peristaltic pump (multidrop combi, Thermo Fisher Scientific, Reinach, Switzerland) equipped with a standard size cassette (tubing inner diameter 1.3 mm, tip inner diameter 0.5 mm) at low speed. After incubation for 1 hour at various temperatures (25, 30, 37, and 41°C), corresponding motilities of 10 replicas were assessed as described above. The average value of absolute movement and corresponding standard deviations, and Wilcoxon rank-sum test for determination of p-values, were calculated in R. The experiment was repeated three times.

### Semi-automated protoscolex motility assay

Several experiments were performed to measure the effects of different compounds (each in six replicas) on the motility of protoscoleces. In an initial experiment, freshly activated protoscoleces (activated by incubation in 0 (DMEM control) to 20% DMSO, pepsin, or pepsin and Na-taurocholate, see above) were tested for their motility as described in this section. In an additional DMSO control experiment, various concentrations of DMSO (0, 0.1, 0.3, 1, 3, and 10%) were incubated with a total number of 20–30 protoscoleces per well in DMEM (without phenol red, containing 10% FCS) for 12 h. Therefore, respective DMSO dilutions were added first to each well and protoscoleces were distributed using the peristaltic pump with the standard size cassette at low speed. The plates were then sealed by pressure sensitive seal and incubated in the incubation chamber of the Nikon live imaging system at 37°C and the motility assessed as described above.

**Fig 1:**
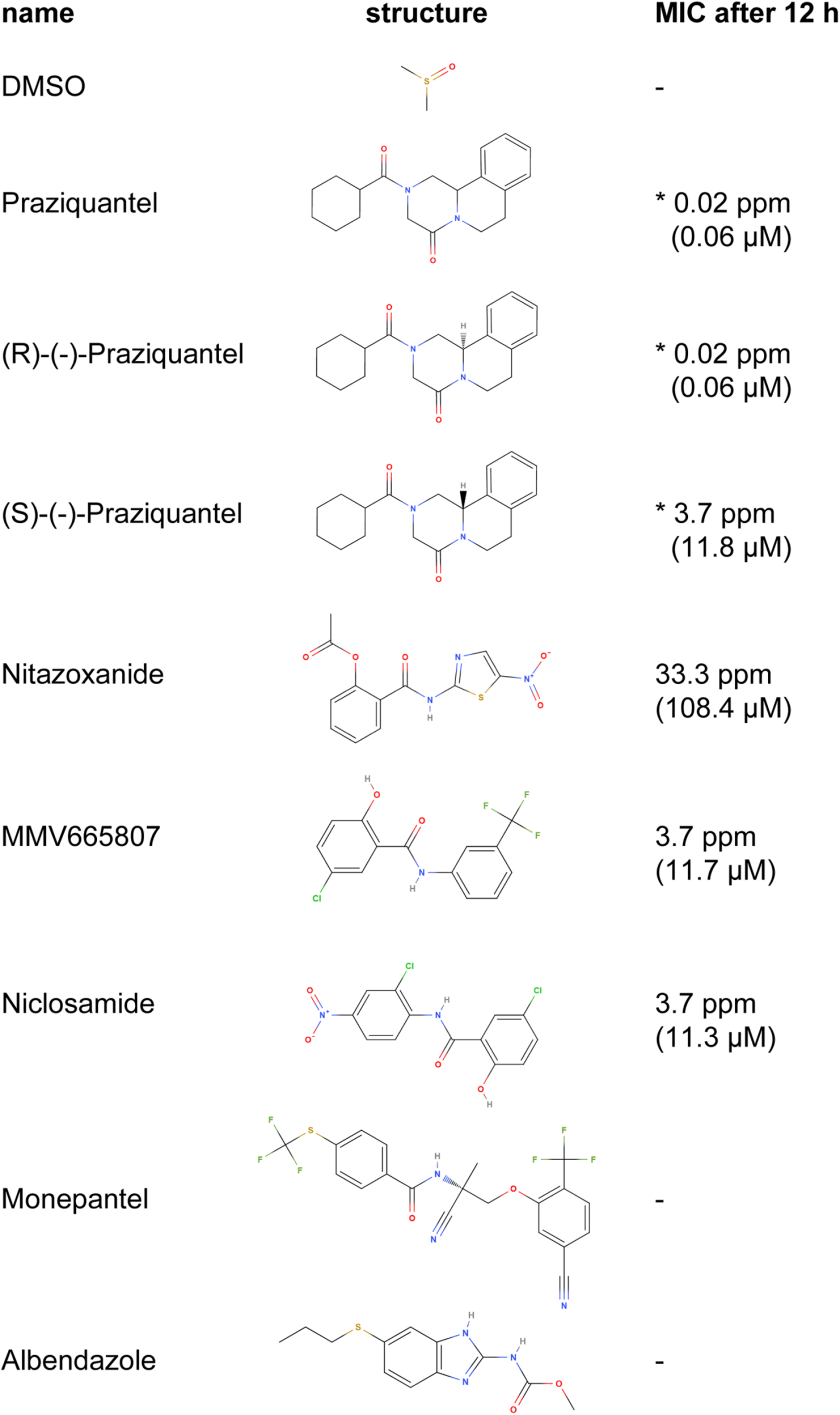
Structures and minimal inhibitory concentrations of drugs tested in the study. Representative minimal inhibitory concentrations (MICs) are given after 12 h of drug incubation. They were determined by calculating the concentration where the slope of the dose-response curve changed significantly and are given in ppm and μM for all drugs (see also Figs 4 and 5). For inactive drugs, no MIC was calculated, (stated by a minus). Asterisks indicate the MICs of PZQ and derivatives that never totally inhibited motility (see Fig 4).

For incubation of activated protoscoleces with various drugs, the compounds were pre-distributed in 5 μL aliquots into 384-well plates as 4x stocks in DMEM (without phenol red, containing 10% FCS and 4% DMSO). To measure the effects of different enantiomers of praziquantel (PZQ), PZQ racemate, (R)-(-)-PZQ and (S)-(-)-PZQ were added to final concentrations of 100 to 0.0006 ppm in a 1:3 dilution series. Further standard compounds (see Fig 1) with known activity (niclosamide [4], nitazoxanide [38]) and known inactivity (albendazole and monepantel), as well as the compound MMV665807 from the open-access malaria box [31], were prepared to final concentrations of 100 to 0.4 ppm in a 1:3 dilution series. Wells with 4% DMSO in DMEM (without phenol red, 10% FCS) served as negative controls. Subsequently 20–30 protoscoleces per well were added in a total of 15 μL DMEM (without phenol red, containing 10% FCS), in order to reach a final DMSO concentration of 1%. They were distributed using a peristaltic pump. Motility was assessed as described above and expressed as percentage of the DMSO control in order to normalize for parasite batch-variations. The experiment with standard compounds was repeated 3 times. The minimal inhibitory concentration (MIC) was determined by calculating the critical concentration where the slope of the dose-response curve changed significantly according to students T-test (p-value < 0.05) in Microsoft Excel 2010.

To evaluate different protoscolex-activation protocols with regards to sensitivity to standard drugs, we compared also the movement of protoscoleces activated with 10% DMSO, pepsin only, or pepsin and Na-taurocholate, and incubated them with the standard compounds as mentioned above.

### Protoscolex viability assessment

In order to compare the effects of the standard drugs as assessed by motility assay also to protoscolex viability, trypan blue staining of protoscoleces was performed. The protoscoleces were extracted and activated as described above. After overnight recovery in DMEM including 10% FCS, the protoscoleces were distributed into 96-well plates of approximately 100 protoscoleces in DMEM without phenol red containing 10% FCS. Subsequently, standard drugs (PZQ, niclosamide, nitazoxanide, albendazole, monepantel and DMSO) and the compound MMV665807 were added to final concentrations of 100 to 0.4 ppm in a 1:3 dilution series with a final DMSO concentration of 1% and in triplicates. The protoscoleces were then incubated with drugs for 18 h at 37°C and 5% CO_2_. For viability staining, trypan blue was added to a final concentration of 0.5% trypan blue during 5 minutes at room temperature. Thereafter, the protoscoleces were washed once in PBS and pictures were taken of each well at 40 times magnification. At last, the trypan blue-stained protoscoleces and the non-stained protoscoleces were counted manually for each picture and the resulting average percentage of viabilities and standard deviations were calculated in Microsoft Excel 2010. This experiment was repeated three times. In order to compare the relative motility of protoscoleces treated with the above mentioned standard drugs to the relative viability of protoscoleces as assessed by trypan blue staining, linear exponential regression was applied in R (version 3.3.2, function lm) for each drug.

### Scanning electron microscopy (SEM)

To compare the morphological effects induced by different activation protocols, or by the drugs PZQ and MMV665807 in more detail, SEM was performed as described by Hemphill and Croft [39]. To compare the morphology of differently activated protoscoleces (DMEM only, 10% DMSO, pepsin, or pepsin and Na-taurocholate), protocols were applied as described above, and protoscoleces were allowed to recover overnight in DMEM including 10% FCS at 37°C, 5% CO_2_. Thereafter, 500 protoscoleces per condition were washed in 0.1 M cacodylate buffer (pH 7.3) and fixed for 2 hours at room temperature in 2 mL glutaraldehyde (2% in 0.1 M cacodylate buffer), before being further treated as described below. To analyze the drug-induced effects on the morphology of protoscoleces, 500 DMSO-activated protoscoleces per well were incubated in a 96 round well plate with 1% DMSO, PZQ (100, 10, 1 and 0.1 ppm, in 1% DMSO) or MMV665807 (100, 10, 1 and 0.1 ppm, in 1% DMSO) in DMEM without phenol red and 10% FCS for 18 hours at 37°C and 5% CO_2_. Thereafter the protoscoleces were washed in 0.1 M cacodylate buffer (pH 7.3) and fixed for 2 hours at room temperature in 2 mL glutaraldehyde (2% in 0.1 M cacodylate buffer). Following three washes in 1 mL of cacodylate buffer (0.1 M, pH 7.3), samples were postfixed in 1 mL osmium tetroxide (2% in 0.1 M cacodylate buffer) during 2 hours, washed twice in water, and specimens were dehydrated in increasing concentrations of ethanol (30, 50, 70, 90, and 3x 100%), and subsequently 1 mL hexamethyldisilazane (HMDS) was added and incubated for 2 minutes. After removal of the HMDS, protoscoleces were resuspended in another 50 μL of HMDS, and were spotted onto a glass coverslip. After evaporation at room temperature, the fixed protoscoleces samples were sputter coated with gold and inspected in a Hitachi scanning electron microscope S-3000 N operating at 25 kV.

### Figure preparation

All figures were prepared in R (version 3.3.2) and formatting as well as compiling was done in Adobe Illustrator (2015.1.0). The compound structures in Fig 1 were depicted by MolView V2.4.

## Results

### Isolation and activation of protoscoleces

**Fig 2:**
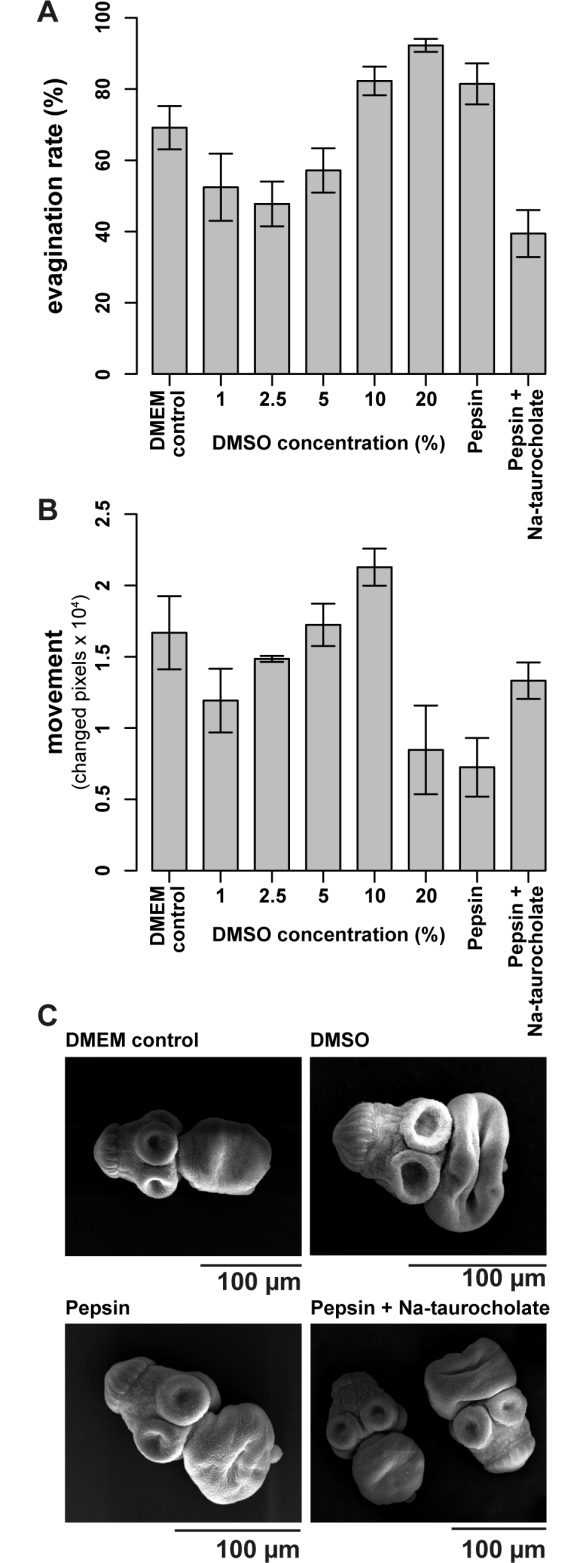
Isolation and activation of protoscoleces for movement-based assay. *E*. *multilocularis* protoscoleces were purified from metacestode material that was grown in gerbils. (A) Subsequent to purification, protoscoleces were evaginated by incubation in various concentrations of DMSO for 3 hours (1, 2.5, 5, 10, and 20%), pepsin (0.5 mg/mL, pH 2) for 3 hours, pepsin (0.05%, pH 2.0, 20 minutes) and Na-taurocholate (0.2%, 3 hours), or in a DMEM control, all performed in triplicates at 37°C. Pictures were taken of each well and the number of in- and evaginated protoscoleces was counted manually. Mean values and standard deviations are given. Highest evagination rates were reached with 20% DMSO. (B) Effects of the induction of evagination on subsequent movement were also analyzed. Mean movement rates (absolute number of changed pixels) and standard deviations are shown. Highest motility rates were measured after activation in 10% DMSO as compared to 20% DMSO, pepsin or pepsin and Na-taurocholate, where motility was reduced. (C) Micrographs of SEM of protoscoleces incubated in DMEM only (control), or activated with 10% DMSO, pepsin only, or pepsin and Na-taurocholate. Based on these pre-trials, evagination was induced by incubation in 10% DMSO in all subsequent experiments. The experiments in A and B were repeated three times and one representative plot is shown each.

Different concentrations of DMSO were applied in addition to the standard method (using pepsin, or pepsin and Na-taurocholate), in order to improve the protocol for evagination and activation of motility of protoscoleces. The isolation procedure itself already led to evagination of 69.1 ± 6.1% of protoscoleces (Fig 2A). Incubation in 20% DMSO for 3 hours yielded the highest (92.3 ± 1.8%) evagination efficiency, while the presence of 1 to 5% DMSO seemed to inhibit rather than promote evagination (Fig 2A). Motility was most pronounced upon incubation in 10% DMSO (2.1 ± 0.1 x 10^4^ changed pixels), and superior to the standard pepsin (0.7 ± 0.2 x 10^4^ changed pixels) or pepsin/Na-taurocholate (1.3 ± 0.1 x 10^4^ changed pixels) methods Fig 2B. Upon activation in 20% DMSO, motility was drastically reduced (0.8 ± 0.3 x 10^4^ changed pixels, Fig 2B). In Fig 2C, SEM micrographs of differently activated protoscoleces are depicted, and they show that the morphology was not affected by the here presented activation methods. A good discrimination of moving and non-moving parasites is crucial for the motility-based drug-screening applied here. Therefore, we concluded to induce evagination and activation by incubation in 10% DMSO for 3 h in all subsequent experiments.

### Assay conditions

**Fig 3:**
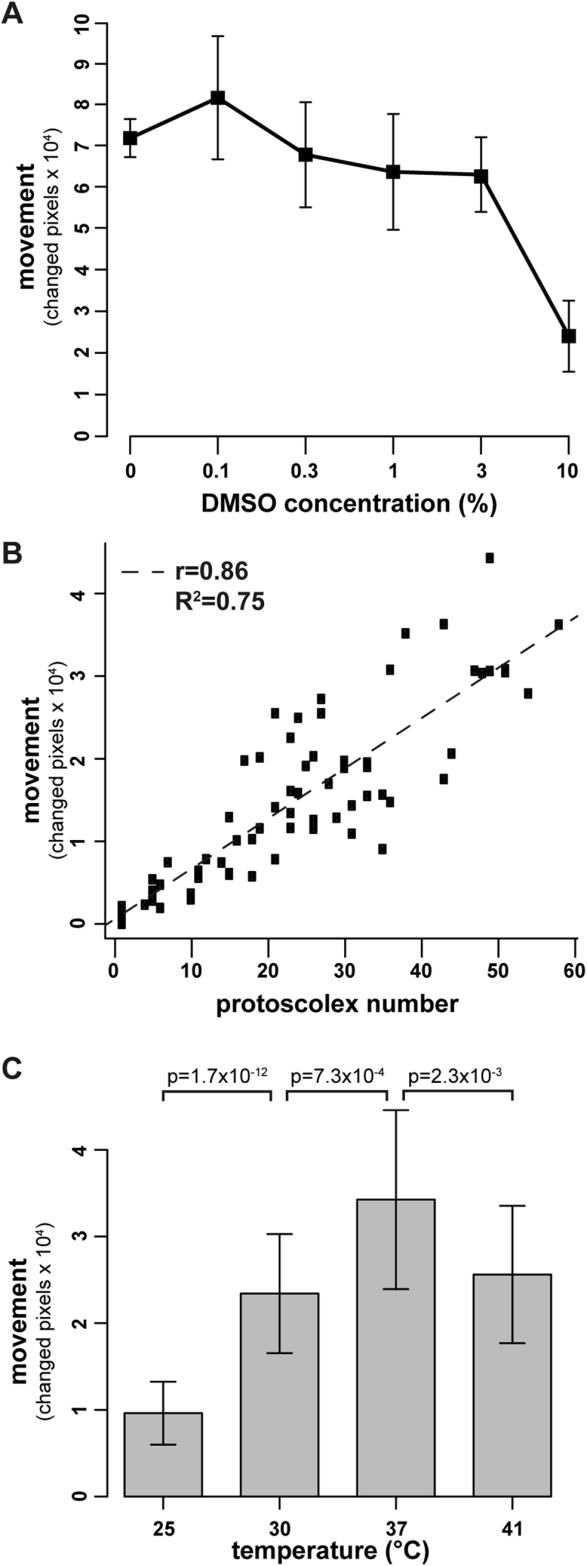
Assay conditions for motility-based assessment of protoscolex movement. *E*. *multilocularis* protoscoleces were purified from metacestodes and activated for 3 h in 10% DMSO. (A) Protoscoleces were distributed into 384-well plates and incubated in various concentrations of DMSO (0, 0.1, 0.3, 1, 3, and 10%) and in 6 replicas each for 12 h, in order to determine the optimal percentage of DMSO to be used for drugs in subsequent tests. The movement per protoscolex was plotted against the respective DMSO concentrations and it shows that protoscolex movement is not significantly reduced up to 3% of DMSO. (B) In order to assess the correlation of protoscolex number per test well and movement, these two parameters were plotted against the total number of protoscoleces per well. A clear linear correlation between number and movement was observed (r = 0.86, R^2^ = 0.75). (C) For determining the optimal assay temperature, protoscoleces were incubated in 1% DMSO at various temperatures (25, 30, 37, and 41°C) with 10 replicas per temperature. As shown, highest movement levels were reached at 37°C, and this was statistically significant. (A-C) The movement of protoscoleces was measured by assessing the changed pixels between two pictures within a 10 seconds interval. (A) and (C) experiments were repeated three times and one exemplary plot is shown.

As all tested drug formulations were dissolved in DMSO, we investigated whether the presence of DMSO would affect the motility of protoscoleces. As shown in Fig 3A, protocoleces incubated in the presence of up to 3% DMSO did not exhibit significantly reduced motility, while protoscoleces incubated in 10% DMSO or higher for 12 h showed largely impaired movement. For practicability of subsequent assays, a final concentration of 1% DMSO was defined for all subsequent drug-testing experiments.

Fig 3B shows the correlation between protoscolex number and movement (r = 0.86, R^2^ = 0.75). For further assays, a representative number of 20–30 protoscoleces per well was chosen, as with this number wells are not overfilled with parasites and individual parasites can be discriminated.

In order to determine the optimal temperature for the assay, comparative runs with protoscoleces incubated for 1 hour in 1% DMSO at different temperatures (25, 30, 37, and 41°C) were performed. As shown in Fig 3C, incubation at a physiological temperature of 37°C yielded the highest motility.

### Effects of PZQ enantiomers on protoscolex motility

**Fig 4:**
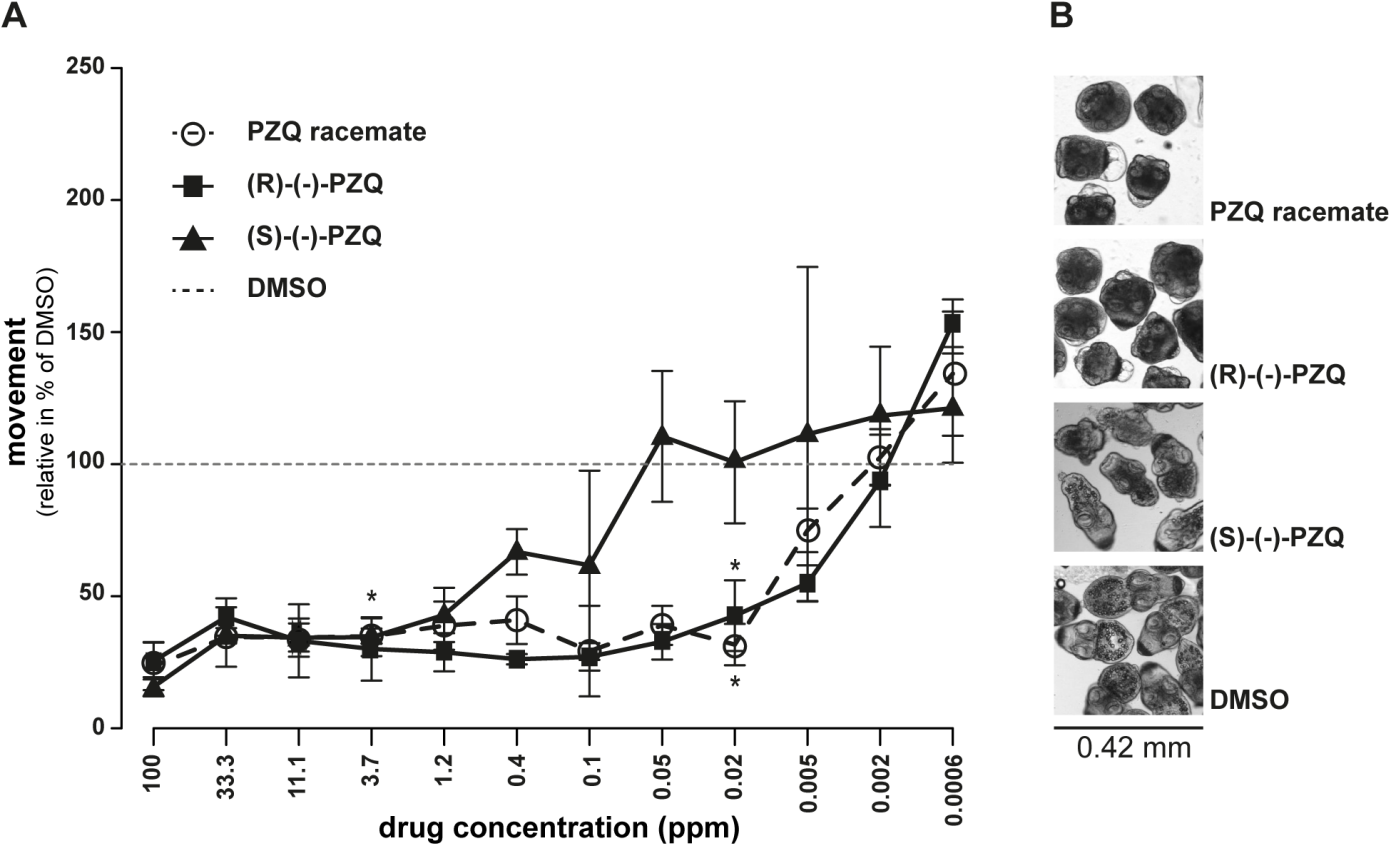
Activity assessment of praziquantel and enantiomers on *E*. *multilocularis* protoscoleces. *E*. *multilocularis* protoscoleces were exposed to various concentrations (100 ppm till 0.0006 ppm, 1 to 3 dilution series) of the PZQ-enantiomers (R)-(-)-PZQ and (S)-(-)-PZQ or the racemic mixture of both (PZQ). After 12 h of incubation, motility was assessed (A) and corresponding pictures of the morphological changes at 0.02 ppm are shown (B). (A) 1% DMSO was set to 100% relative movement and all other drug-responses were calculated accordingly. Note that all forms of PZQ never led to a complete inhibition of protoscolex movement. (R)-(-)-PZQ and the racemic mixture exhibited higher activities than the (S)-(-)-PZQ, with minimal inhibitory concentrations (MICs) indicated by asterisks (see Fig 1 for compound structures and S1 Fig for other measurement time points). Experiments shown here were repeated three times.

For a first validation of the test, the standard drug in use (PZQ) as well as its R- and S-enantiomers were tested in the protoscolex motility assay. As shown in Fig 4A and S1 Fig, neither the racemic mixture of PZQ, nor its enantiomers (R)-(-)-PZQ or (S)-(-)-PZQ led to complete inhibition of motility at concentrations up to 100 ppm and up to 24 h of incubation time. After 1 h, drug-induced effects did not yet reach their maximum, but as shown in S1 Fig, after 12 h the maximum effects were largely reached. Therefore, in all subsequent experiments, 12 h drug incubations were applied. Based on the drug-response curves of 12 h of drug-incubation, the MICs were determined: (R)-(-)-PZQ and the racemic mixture of PZQ had a MIC of 0.02 ppm (0.06 μM), whereas (S)-(-)-PZQ had a MIC of 3.7 ppm (11.8 μM) (Fig 1). Corresponding micrographs showing morphological changes induced by each drug at 0.02 ppm after 12 h of drug incubation are depicted in Fig 4B. Movies for comparison of effects of PZQ as compared to DMSO are shown in S1 Movie.

### Effects of anti-parasitic drugs on protoscolex motility

**Fig 5:**
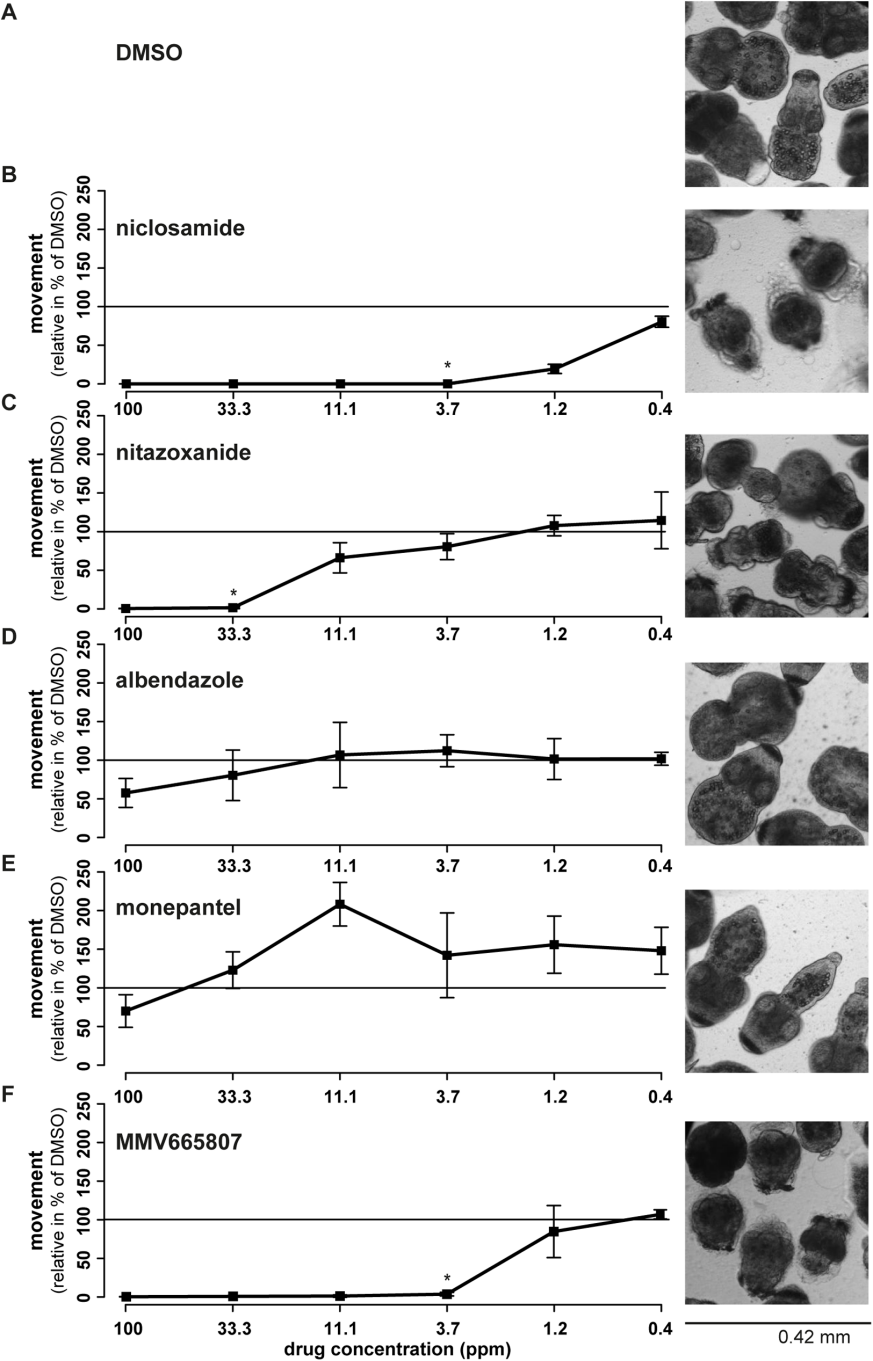
Activity assessment of various drugs against *E*. *multilocularis* protoscoleces. Various standard compounds with described activity (niclosamide (B) and nitazoxanide (C)) or no activity (albendazole (D), monepantel (E)) against adult cestodes were assessed concerning their effects on *E*. *multilocularis* protoscolex movement (100 to 0.4 ppm, 1 to 3 dilution series) and morphology (shown for 33.3 ppm). Protoscoleces treated with 1% DMSO (A, solvent control) were set to 100% relative movement and all other drug-incubations were calculated accordingly. Each drug was assessed in 6 replicas after 12 h of drug-incubation and mean percentages and standard deviations are given. Further time points are given in S2 Fig, compound structures and MICs (as indicated here by asterisks) are given in Fig 1. In addition, also effects of the novel compound, MMV665807, were assessed (F) by means of impact on motility and morphology. Mean percentages of movement and standard deviations are given. The MIC is indicated by an asterisk. Further time points are given in S2 Fig. All these experiments were repeated at least 3 times.

Additional reference compounds with known activity against adult cestodes or protoscoleces (niclosamide and nitazoxanide) and without activity against adult cestodes or protoscoleces (albendazole and monepantel) were selected, in order to further validate the protoscolex motility assay. Also for the use of these standard compounds, maximal drug-effects were reached after 12 h of incubation on (see S2 Fig). Therefore, results corresponding to the 12 h time points are provided in Fig 5. One exception is nitazoxanide that, interestingly, lost its activity slightly over time. Niclosamide and nitazoxanide reduced the motility of protoscoleces in a dose- and time-dependent manner (Fig 5A–5E, S2 Fig). Morphological effects induced at a drug concentration of 33.3 ppm are shown in Fig 5A–5E. Corresponding MICs are given in Fig 1. Both albendazole and monepantel did not inhibit the motility of parasites at any of the concentrations tested, except some slight reduction in motility at 100 ppm. We also compared the above described standard drugs against protoscoleces that were activated by 10% DMSO, pepsin, or pepsin and Na-taurocholate. As shown in S3 Fig, protoscoleces followed the same drug-responses independent of which activation method was used. However, differences between active and inactive drugs were highest when protoscoleces were activated with DMSO, as visible in the absolute movement data (S3 Fig).

In addition, a compound previously described to exhibit parasiticidal activity against the metacestode stage of *E*. *multilocularis*, MMV665807 [31], was assessed for its motility-reducing activity on protoscoleces. As shown in Fig 5F (and S2 Fig), the drug induced a strong inhibition of motility with a MIC of 3.7 ppm (11.7 μM) after 12 h of drug incubation. Corresponding morphological effects (at 33.3 ppm) are visualized in Fig 5F and a representative movie is provided in S1 Movie.

### Effects of anti-parasitic drugs on protoscolex viability

**Fig 6:**
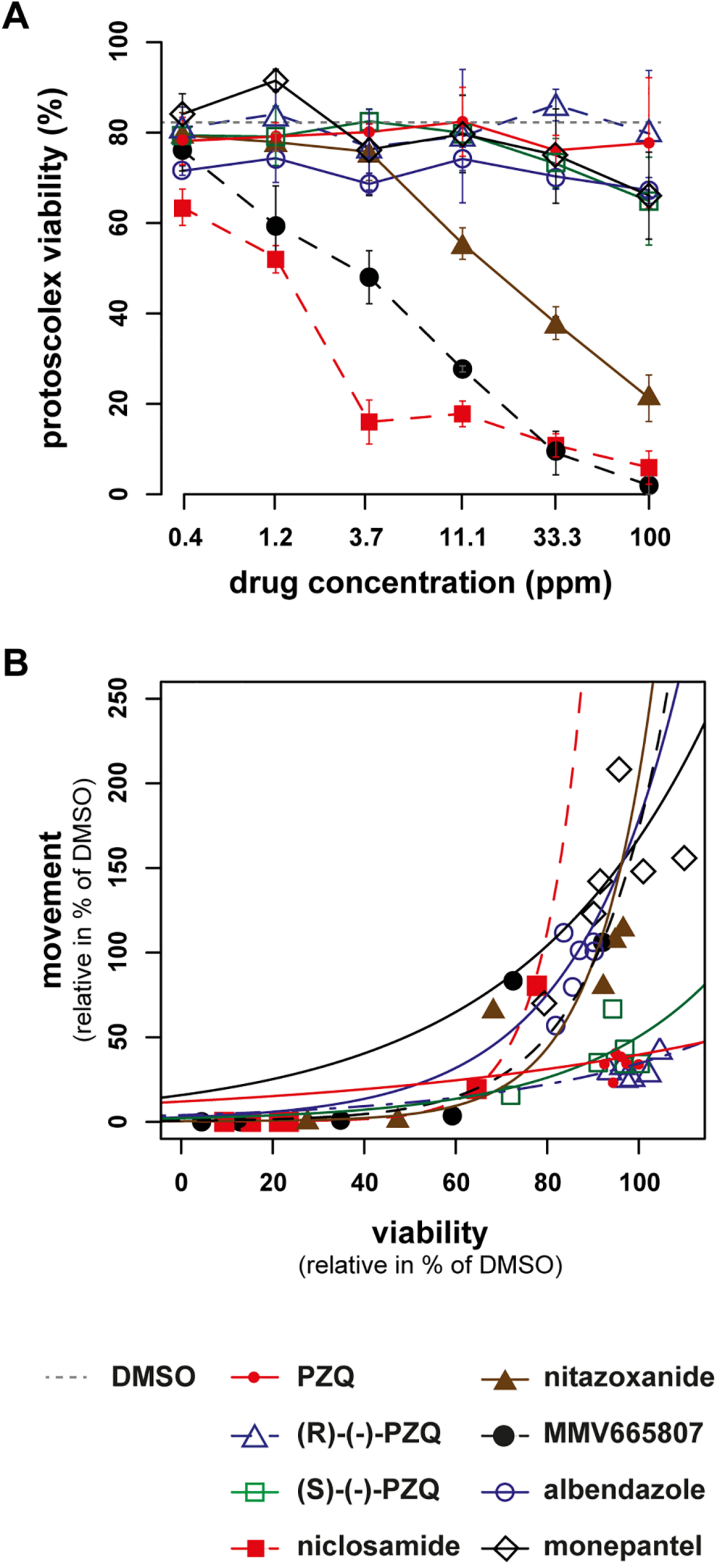
Assessment of viability of *E*. *multilocularis* protoscoleces treated with various drugs. (A) Trypan blue staining of protoscoleces was performed after 12 h of drug-incubation in triplicates (PZQ and enantiomers, niclosamide, nitazoxanide, MMV665807, albendazole, monepantel, concentrations from 100 to 0.4 ppm, 1 to 3 dilution series) and the mean percentages and standard deviations of protoscolex viabilities are shown. Protoscoleces incubated in the solvent DMSO at 1% served as control (dotted line). PZQ did not reduce the viability of protoscoleces, whereas niclosamide, nitazoxanide and MMV665807 did. The experiment was repeated 3 times and one exemplary plot is shown. (B) shows the correlation between effects on motility and viability (both in % of DMSO) for differently treated protoscoleces after 12 h. The correlation follows an exponential curve for all active drugs except PZQ. Respective functions and R^2^ values are given in S1 Table. The same legend applies for both subfigures.

For comparative reasons, all drugs described above were also assessed by trypan blue staining, which is the standard method of protoscolex viability testing (Fig 6A). The viability of protoscoleces was dose-dependently lowered upon incubation with niclosamide, MMV665807 and nitazoxanide as shown in Fig 6A. The drugs albendazole and monepantel had only slight effects on the viability of protoscoleces. The drug currently used for therapy, PZQ, as well as its enantiomers, showed only minor effects on the viability of protoscoleces at all tested concentrations. The correlation between motility and viability followed an exponential correlation curve for all active drugs except PZQ (Fig 6B and S1 Table for functions and R^2^ values). Interestingly, the motility of protoscoleces had already been completely inhibited, when viability was still at 50%, which highlights that a reduced motility does not necessarily indicate a reduction or loss of viability. PZQ and its enantiomers did not follow an exponential correlation curve, as they never killed the protoscoleces (Fig 6B).

### Morphological changes observed by SEM

**Fig 7:**
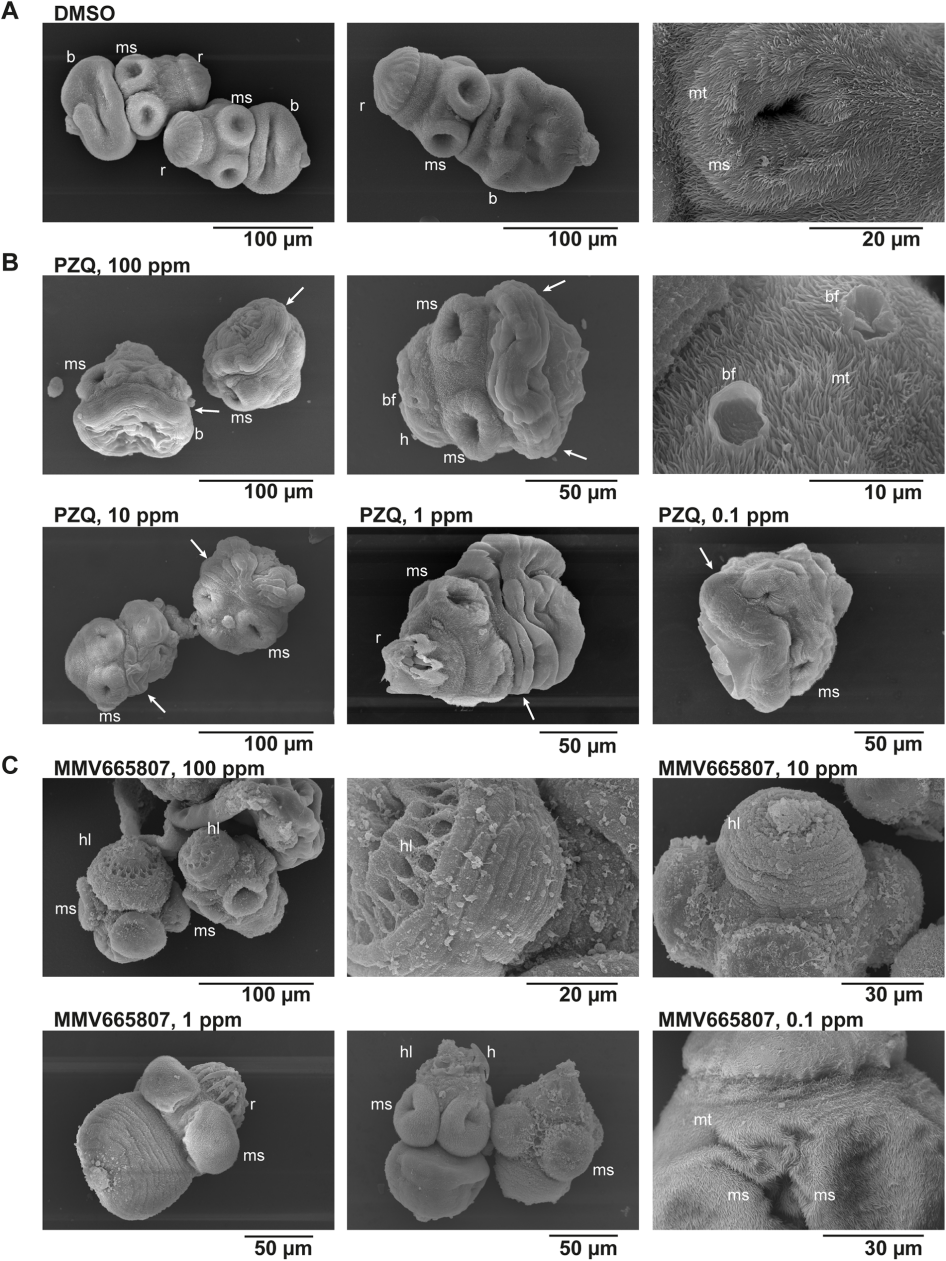
Scanning electron microscopical assessment of protoscoleces treated with praziquantel and MMV665807. Protoscoleces were incubated in (A) 1% DMSO (control), (B) praziquantel (PZQ), or (C) MMV665807 (the latter two both with 1% DMSO) for 12 hours at 100, 10, 1 and 0.1 ppm and subsequently subjected to scanning electron microscopy (SEM). (A) depicts intact protoscoleces with rostellum (r), including hooks covered by a filamentous network, four muscular suckers (ms) and the protoscolex body (b). The whole protoscolex is covered in microtriches (mt) as shown in the enlarged view of a muscular sucker on the right. (B) Noticeable effects of PZQ on protoscoleces were seen at all concentrations tested (contraction of body and neck region (indicated by arrows), bleb formation (bf), as magnified in the picture most to the right), but the muscular suckers, hooks (h) and microtriches were largely unaffected. (C) MMV665807 led overall to less contraction compared to PZQ, but it induced a striking loss of microtriches and hooks from the rostellum (hl, see especially second picture at 100 ppm) at high concentrations (100, 10 and partially 1 ppm). At low concentrations (0.1 ppm), microtriches and rostellum with hooks were fully intact.

The standard drug PZQ and the newly identified MMV665807 were also compared regarding their effects on the morphology of protoscoleces. Control protoscoleces that were incubated in 1% DMSO showed the characteristic structures of body and head with rostellum and 4 muscular suckers. The protoscolex surface was comprised of microtriches (Fig 7A). All PZQ-treated protoscoleces showed the same effects, regardless of the tested concentration (100 to 0.1 ppm): they were contracted, especially in the neck region, and started forming blebs all over the body. The microtriches, suckers and the rostellum were not visually affected by PZQ (Fig 7B). Treatment with a concentration of 100 and 10 ppm of MMV665807 (Fig 7C) also led to contraction of the protoscolex body, but to a lower extent compared to PZQ. In addition, the microtriches and the tissue mesh covering the rostellum were both lost and therefore the hooks were not present anymore. The suckers were not visibly affected by MMV665807. At 1 ppm, MMV665807 showed still some of these effects, but at 0.1 ppm, all protoscoleces were visually indistinguishable from the control sample. These morphological findings further confirmed the loss of activity of MMV665807 at concentrations lower than 3.7 ppm (Fig 5).

## Discussion

The present paper describes for the first time an *in vitro*-based screening test that will allow researchers to evaluate compounds for activity against adult cestodes. In contrast to most other species, *E*. *multilocularis* multiplies asexually in its intermediate hosts. Therefore, the generation of parasite material for this test is easy, as metacestode-containing protoscoleces are usually available in excess in laboratories maintaining *E*. *multilocularis* in gerbils. The purification of protoscoleces from metacestodes is simple and straight forward and allows high numbers of protoscoleces to be purified from a batch of metacestode material originating from one euthanized animal.

In this assay, motility is used as a phenotypic readout of worm activity, which is often, but not always, coincidental with viability. A reduction of motility to zero could theoretically be seen in a worm that is still alive. However, a viable worm that cannot move, or is severely limited in movement, will be expelled from the gastrointestinal tract [40–42]. Therefore, we consider motility as a suitable readout for the screening of new drugs against adult cestodes. We induced evagination and motility by incubation in DMSO, and did not follow the standard pepsin or pepsin/bile acid methods. DMSO-activation resulted in higher resolution of different motility rates, did not alter the drug-response, did not induce any visible damage to the parasite, and, from the technical point of view, DMSO activation is easy and highly reproducible, since no pH adjustments are necessary. In addition, we also compared the quantified readouts of the motility assay to the subjective and laborious trypan blue viability staining, and we found an exponential correlation between the methods. The lag phase of this correlation curve highlights that inhibition of motility does not necessarily lead to loss of viability.

Described targets of PZQ are voltage-gated calcium-channels and adenosine receptors, both leading to an influx of calcium ions that induces paralysis in the worm [7]. Thus, reduction of protoscolex movement by PZQ-treatment was expected. Our tests revealed that drugs with known anthelmintic activity (PZQ, niclosamide and nitazoxanide) were active and reduced motility of protoscoleces, and those known to be ineffective against adult *Echinococcus* (albendazole and monepantel) were not. For PZQ we determined a MIC of 0.02 ppm, which is in line with previous observations on *Echinococcus* protoscoleces [43,44] as well as a variety on various adult cestodes [45,46]. Niclosamide was described to be active against *Hymenolepis nana* adults at 0.1 ppm within 30 minutes [46], and we observed a slightly higher MIC of 3.7 ppm against *E*. *multilocularis* protoscoleces. Nitazoxanide showed a MIC of 33.3 ppm after 12 h against *E*. *multilocularis* protoscoleces, which is comparable to results found earlier against *E*. *granulosus* protoscoleces (5 ppm after 3 days [47]), as well as the general activity of nitazoxanide against a variety of nematodes with MICs ranging between 10 to 100 ppm [48,49].

Treatment with PZQ led only to a partial paralysis of *E*. *multilocularis* protoscoleces. This observation has, to the best of our knowledge, been unknown so far. Viability assessment by trypan blue further confirmed this finding, as PZQ reduced the viability only slightly. Interestingly, after one hour of drug-incubation, PZQ rather increased parasite motility, which could be a first sign of stress response towards the drug. Further, the motility assay allowed also to discriminate between the activity of PZQ enantiomers, and (R)-(-)-PZQ was confirmed as the more active enantiomer, as previously reported for *Schistosoma* [50]. However, it cannot be concluded whether the (S)-(-)-PZQ has some slight intrinsic activity, or whether this activity simply resulted from impurities in the enantiomer preparation (purity stated by the manufacturer ≥ 95%). In addition, the electron microscopical assessments revealed clear morphological alterations in protoscoleces treated with PZQ. These changes were largely in line with previously described effects of the drug when applied on adult *Echinococcus* worms, as also in protoscoleces immediate contraction, neck shortening and bleb formation were observed [7,51]. In contrast to descriptions for adult worms, hooks were only partially lost and the suckers did not expand into a convex shape in the present study [51].

One interesting new compound identified by this novel assay: MMV665807, a derivative of niclosamide that is provided within the MMV malaria box [52]. MMV665807 showed a MIC in the same range as niclosamide, and it reduced the viability of protoscoleces to the extent that is comparable to niclosamide. Future *in vivo* confirmatory studies, such as in the *Hymenolepis* mouse model, will show, whether the compound exhibits also good *in vivo* anti-cestode activity. Electron microscopical assessment of protoscoleces treated with MMV665807 showed a clear difference to the changes observed by PZQ, which implies that another mode of action is involved. The mode of action of MMV665807 against *E*. *multilocularis* is currently under investigation.

Motility assessment by comparison of change in pixels of two single pictures over a 10 seconds interval was chosen, as such a simplified setup reduces the computational power and analytical complexity needed for data analysis. Previous publications on other motility-based tests of helminths used to employ video-based analyses (e.g. [22]), but due to their analytical complexity they are not easily transferable to higher throughput assays [13]. An approach that could be of interest for increased throughput is the cheaper model described by Marcellino et al [20], where a whole test plate is measured at once. However, in this assay only 24-well plates were applied, as the filarial worms tested with this approach were too large to allow for smaller culture devices. Another approach, based on a real-time monitoring device from Roche, was used for *Haemonchus contortus*, *Strongyloides ratti*, hookworms and blood flukes and it allows entire plate assessments as well [17]. However, this device is expensive and so far also restricted to the 96-well format. The more simple Wormscan is relatively cheap, but restricted to the 12-well format [21]. Microfluidic-based platforms for screening of anthelminthics and resistance allow the in-depth and real-time study of the response of worms to drugs, and are therefore of high interest for the study of selected compounds [53]. However, none of the mentioned assays was shown to be applicable for the screening of adult cestodes. Another study by Camicia et al. (2013) showed that *E*. *granulosus* protoscolex motility can be monitored in the worm tracker method developed for *C*. *elegans* by Simonetta et al. [54], and they also showed that the activity of the neurotransmitter inhibitor citalopram could be detected by this system [55]. Any further application of this system on *Echinococcus* protoscoleces has, to the best of our knowledge, not been published and the test has also not been applied as a cestode-screening system. An alternative approach that could be used for larger-scale screening of drugs against adult cestodes, and anthelmintics in general, would be stem cell-based. For *E*. *multilocularis*, such an assay would theoretically be feasible, as stem cell cultivation [28] and drug testing on stem cells [31] has already been implemented. However, compared to the simplicity of protoscolex generation and purification, as well as the presence of intact parasite structures in the protoscolex-based approach, we consider the whole-organism protoscolex screening to be more suitable.

In conclusion, the screening assay described in this paper is based on protoscoleces of *E*. *multilocularis*. Protoscoleces are sufficiently small to allow testing in 384-well format with multiple parasites per well and the setup is inexpensive. Furthermore, it can basically be carried out with any microscope that allows digital images to be taken, thus no highly specialized equipment is needed. The motility-based assay will allow objective and medium-throughput screening of substances against cestodes, and at the same time enables researchers to visualize morphological effects. As such, the motility-based assay will be further applied for the testing of drug libraries, novel compound classes, and for the in-depth characterization of MMV665807, a new compound of interest with respect to activity against adult cestodes.

## Supporting information

S1 File JOBS module applied for automated picture taking of protoscoleces in NIS elements version 4.40 (Nikon). (BIN)

S2 File Macro for image-based motility assessment in ImageJ version 1.49. (TXT)

S1 Movie Short movies of protoscoleces incubated with DMSO (1%) or 10 ppm of praziquantel, albendazole or MMV665807. (MP4)

S1 Fig Time course of activity of praziquantel (PZQ), (S)-(-)-PZQ and (R)-(-)-PZQ on protoscoleces at six concentrations (100, 33.3, 11.1, 3.7, 1.2, and 0.4 ppm) at various time points (1, 6, 12, 18, and 24 h). (TIF)

S2 Fig Time course of activity of niclosamide, nitazoxanide, albendazole, monepantel and MMV665807 on protoscoleces at six concentrations (100, 33.3, 11.1, 3.7, 1.2, and 0.4 ppm) at various time points (1, 6, 12, 18, and 24 h). (TIF)

S3 Fig Activity assessment of standard drugs against *E*. *multilocularis* protoscoleces that were activated by DMSO (10%, 3 hours), pepsin (0.5 mg/mL, pH 2, 3 hours), or pepsin (0.05%, pH 2, 20 minutes) and Na-taurocholate (0.2%, 3 hours). A) shows relative movement values in relation to DMSO (100%), B) depicts absolute movement values for each drug.
(TIF)

S1 Table Functions and R^2^ values of the exponential correlation curves given in Fig 6. (XLS)
