# Supplementary figures and images for: Development of a movement-based in vitro screening assay for the identification of new anti-cestodal compounds
Source: PLoS Negl Trop Dis. 2017 May 17;11(5):e0005618. doi: 10.1371/journal.pntd.0005618 (PMC5448807; doi:10.1371/journal.pntd.0005618)

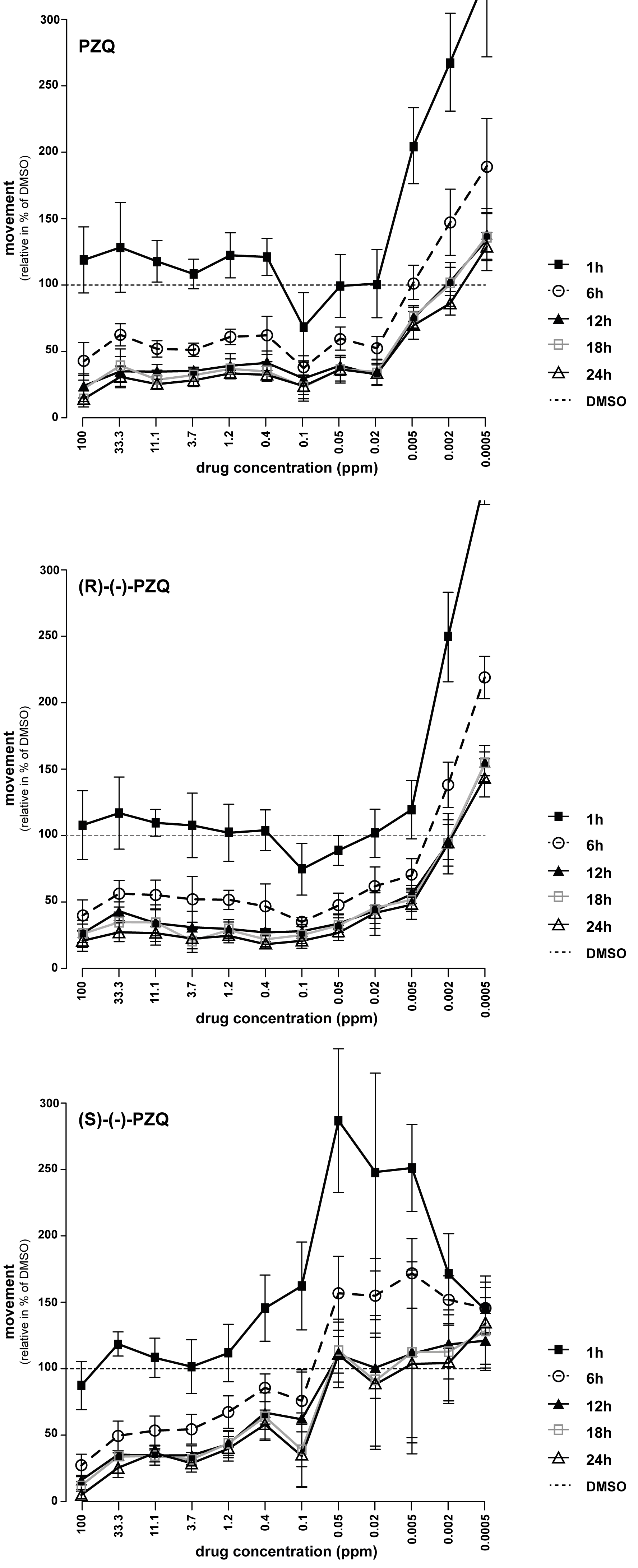

Supplement: S1 Fig — (TIF) [file pntd.0005618.s004.tif]

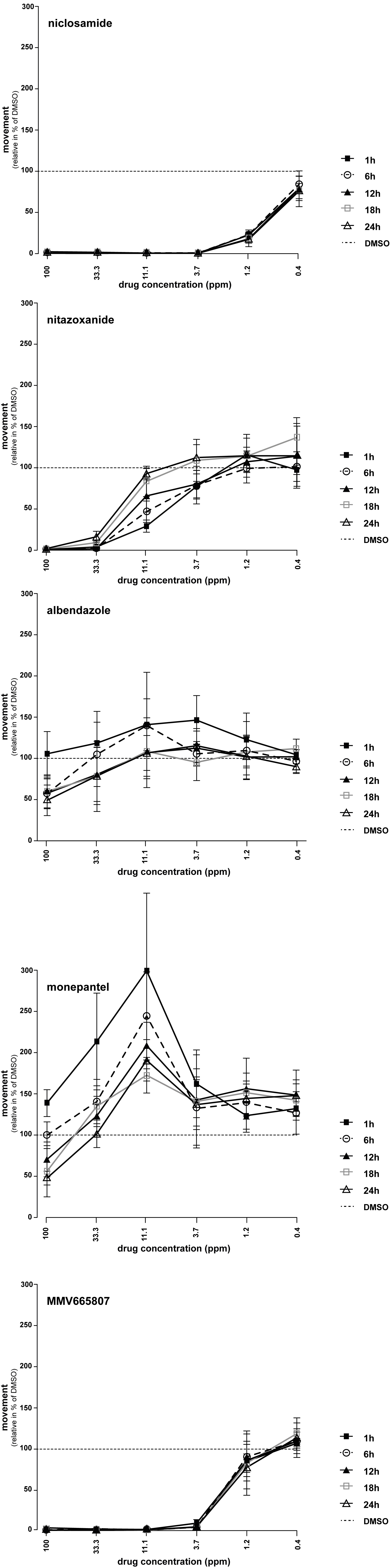

Supplement: S2 Fig — (TIF) [file pntd.0005618.s005.tif]

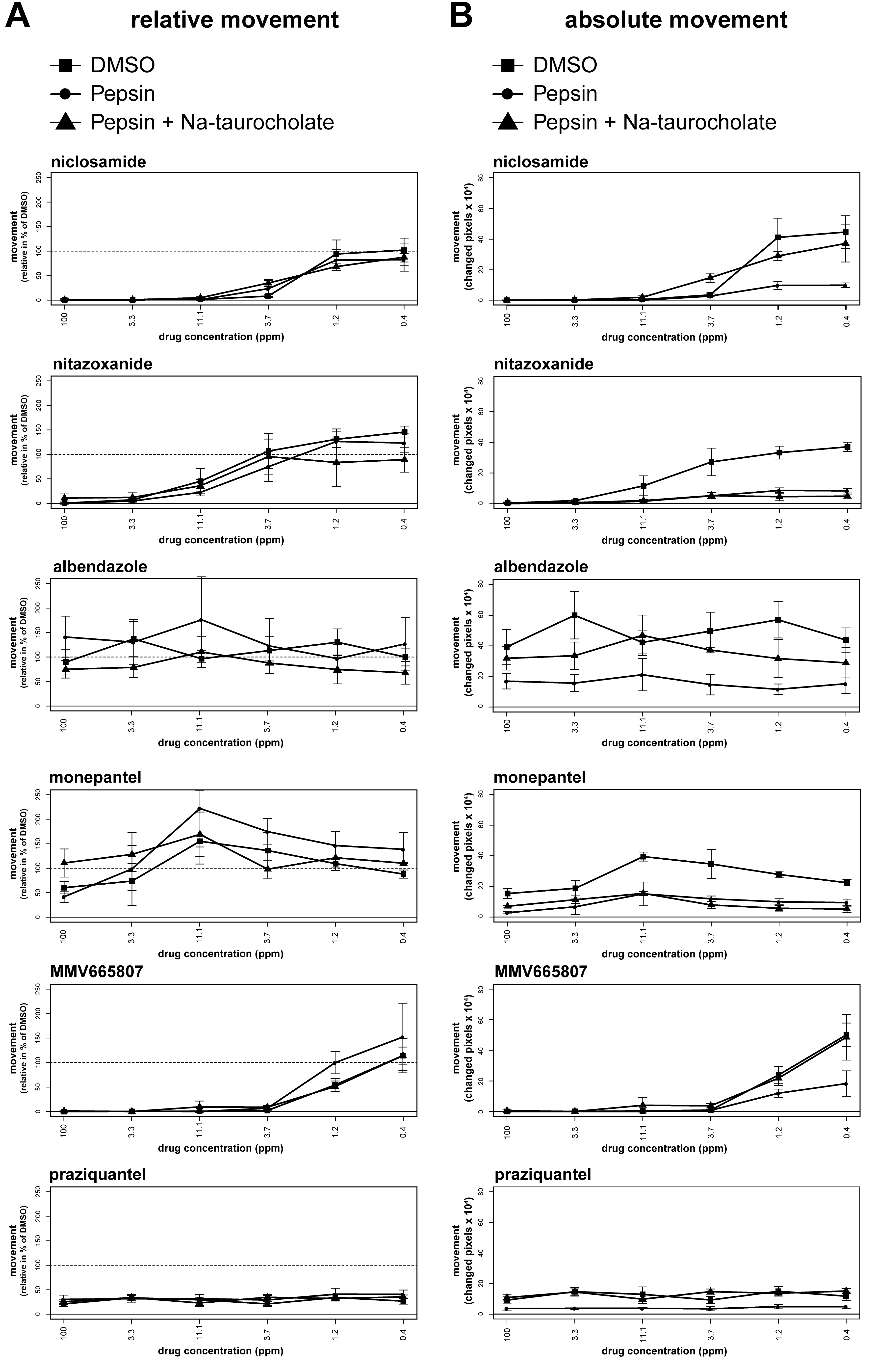

Supplement: S3 Fig — A) shows relative movement values in relation to DMSO (100%), B) depicts absolute movement values for each drug. (TIF) [file pntd.0005618.s006.tif]
